# Acetylation‐like H2BK109Q attenuates acute brain injury and preserves axon‐myelin integrity after intracerebral hemorrhage

**DOI:** 10.1002/cns.71172

**Published:** 2026-10-09

**Authors:** Sinan Jin, Meng Yang, Jincheng Meng, Ziyi Han, Yousef Rastegar‐Kashkooli, Pengtian Lu, Junmin Wang, Jian Wang, He Wu

**Affiliations:** ^1^ Department of Pathology The First Affiliated Hospital of Harbin Medical University Harbin China; ^2^ State Key Laboratory of Frigid Zone Cardiovascular Disease Harbin China; ^3^ College of Medical Laboratory Technology Harbin Medical University Harbin China; ^4^ Department of Human Anatomy, School of Basic Medical Sciences Zhengzhou University Zhengzhou China; ^5^ Aging Decoding and Regeneration Institute, School of Basic Medical Sciences Nanozyme Laboratory in Zhongyuan State Key Laboratory of Antiviral Drugs Pingyuan Laboratory Zhengzhou University Zhengzhou China

**Keywords:** axon‐myelin injury, galactosylceramidase, histone H2B, intracerebral hemorrhage, lysine acetylation, sphingolipid metabolism

## Abstract

**Aims:**

Axon–myelin damage contributes to neurological dysfunction after intracerebral hemorrhage (ICH), but the upstream chromatin regulators remain unclear. We aimed to characterize the H2B acetylation response to ICH, test the functional significance of modeled H2BK109 states, and identify a downstream effector of H2BK109Q‐mediated protection.

**Methods:**

Pan‐H2B acetylation was examined in mice with collagenase‐induced ICH and in hemoglobin‐treated HT22 cells, and the histone acetylome of the mouse brain was profiled. H2BK109Q and H2BK109R were used to model acetylation‐mimetic and non‐acetylated lysine‐like states. Injury outcomes, axon–myelin integrity, molecular profiles, and regional Galc knockdown were assessed.

**Results:**

ICH and hemoglobin reduced pan‐H2B acetylation, and acetylome profiling indicated reduced H2BK109ac. H2BK109Q‐expressing mice showed higher pan‐H2B acetylation, smaller hematomas, less edema and apoptosis, improved neurological function, and greater preservation of LFB, MBP, NF200, and axon–myelin ultrastructure. Under matched hemoglobin exposure, H2BK109Q‐expressing HT22 cells showed higher BCL2/BAX ratios and lower TUNEL positivity than H2BK109R‐expressing cells. In vitro analyses highlighted GALC as a candidate downstream effector of H2BK109Q‐associated protection. In H2BK109Q‐expressing mice, Galc knockdown reduced LFB‐ and MBP‐positive areas and NF200 signals in the caudate putamen and corpus callosum compared with untreated and siNC controls.

**Conclusion:**

H2BK109Q attenuates acute ICH injury and preserves axon–myelin integrity, with GALC contributing to these protective effects.

## Graphical abstract

After intracerebral hemorrhage, reduced pan‐H2B acetylation is linked to acute brain injury and axon–myelin damage. The acetylation‐mimetic H2BK109Q substitution attenuates injury and preserves axon–myelin integrity. Regional Galc knockdown diminishes this protection, identifying GALC as a primary contributing effector.

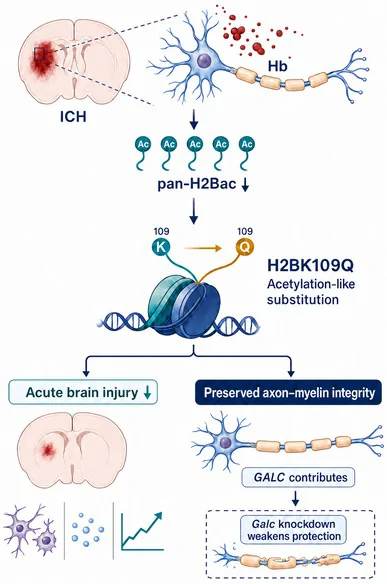

## 1 Introduction

Intracerebral hemorrhage (ICH) remains a devastating stroke subtype with high mortality and persistent disability. The hematoma causes immediate tissue disruption, whereas blood‐derived products, inflammatory mediators, and immune‐mediated blood–brain barrier disruption contribute to perihematomal injury [1, 2, 3, 4]. Perihematomal edema is clinically relevant, although its association with outcome depends on the timing and method of measurement; recent clinical evidence further indicates that peak perihematomal edema expansion is independently associated with poor functional outcome [5, 6, 7]. ICH is therefore an evolving tissue injury in which early molecular responses can shape structural and neurological damage.

Axons and myelin are particularly vulnerable to this evolving hemorrhagic brain injury. Their disruption impairs long‐range neural connectivity and contributes to neurological dysfunction. Single‐cell and experimental studies have shown coordinated responses among neuronal, oligodendroglial, and myeloid cells in hemorrhagic white matter [8, 9]. Interventions that preserve axons and myelin improve functional recovery in ICH models [10, 11]. However, the upstream chromatin events that influence acute axon–myelin vulnerability remain poorly characterized.

Epigenetic regulation may link hemorrhagic stress to coordinated cellular responses. Experimental ICH alters chromatin accessibility across brain cell populations and shifts regional acetylation of H3/H4 [12, 13]. Site‐specific histone lactylation can either exacerbate neuronal death or support microglia‐associated white matter responses, depending on the residue and cellular context [14, 15]. Histone deacetylase inhibition also reduces experimental ICH injury and preserves white matter [16, 17]. Because these interventions affect many histone and nonhistone substrates, they cannot pinpoint the residue responsible for protection.

Histone H2B is a strong candidate for residue‐level analysis but has received less attention than H3 and H4 in the context of brain injury. H2B acetylation marks active regulatory regions and can persist through mechanisms beyond transcriptional by‐products [18, 19]. H2B modification also participates in apoptotic chromatin responses [20]. In this study, we observed an early decrease in pan‐H2Bac in mice with collagenase‐induced ICH and in Hb‐treated HT22 cells. Brain acetylome profiling further indicated reduced H2BK109ac. H2BK109Q increased pan‐H2Bac in mouse brain tissue, reduced hematoma volume and brain edema, improved neurological function, and attenuated apoptosis. Moreover, GALC contributed to H2BK109Q‐mediated preservation of axon–myelin integrity after ICH.

## 2 Materials and Methods

### 2.1 Animals and Experimental ICH

Eight‐week‐old male C57BL/6 mice (approximately 22 g; Changsheng Biotechnology, Liaoning, China) were housed under controlled temperature and humidity on a 12 h light/dark cycle, with free access to food and water. Mice were randomly assigned to experimental groups. Only male mice were used to minimize variation associated with estrogen‐related neuroprotection [21]. All procedures were approved by the Ethics Review Committee for the Use and Welfare of Experimental Animals of the First Affiliated Hospital of Harbin Medical University (IACUC No. 2022132) and were conducted and reported in accordance with ARRIVE 2.0 [22].

ICH was induced by stereotaxic injection of collagenase VII‐S into the left striatum, based on established mouse ICH procedures [23, 24]. Under isoflurane anesthesia, the microsyringe needle was positioned 1.0 mm anterior and 2.2 mm lateral to bregma, advanced to 2.7 mm below the skull surface, and then withdrawn to 2.6 mm for collagenase infusion. Collagenase VII‐S (C0773, Sigma‐Aldrich; 0.075 U/μL; 0.4 μL; total dose, 0.03 U per mouse) was infused into the left striatum. Sham mice received 0.4 μL of saline using the same procedure (Figure S1A,B). Brain tissue was collected at the prespecified time points. Full surgical procedures are provided in the Supplementary Methods.

### 2.2 H2BK109Q and H2BK109R AAV Constructs and GALCsiRNA Delivery

The lysine‐109 codon of mouse *H2bc18* (AAA) was replaced with CAA (H2BK109Q) or CGT (H2BK109R). AAV2/9 vectors expressed 3 × Flag‐tagged H2BK109Q or H2BK109R together with ZsGreen, or ZsGreen alone, under the CMV promoter. H2BK109Q and H2BK109R served as acetylation‐mimetic and non‐acetylated lysine‐mimetic substitutions, respectively.

Each AAV was injected at 2 μL into the left striatum 21 days before ICH. For GALC loss‐of‐function experiments, 100 pmol of GALC‐specific siRNA (siGALC) or negative‐control siRNA (siNC) in 2 μL was injected into the same ipsilateral striatal region 24 h before ICH. GALC protein levels were measured in the injected region. The experimental timeline is shown in Figure 1A; vector details, titers, siRNA sequences, and injection procedures are provided in the Supplementary Methods.

### 2.3 HT22 Cell Culture and Hb Exposure

Mouse hippocampal neuron‐like HT22 cells (iCell Bioscience, Shanghai, China) were cultured in Dulbecco's Modified Eagle Medium supplemented with 10% fetal bovine serum, 100 μg/mL streptomycin, and 100 U/mL penicillin at 37°C in 5% CO2. Stable ZsGreen control, H2BK109R, and H2BK109Q cell populations were generated by lentiviral transduction followed by puromycin selection.

Hb exposure was used to model controlled blood‐derived neuronal injury [25, 26]. Time‐ and concentration‐response experiments showed a pronounced nadir in pan‐H2Bac after exposure to 10 μmol/L Hb for 12 h; this condition was therefore used as the standardized Hb challenge for subsequent comparisons. Con cells were untransduced, whereas NC cells carried the ZsGreen control vector without an H2B insert. All comparison groups received the same Hb exposure.

### 2.4 Histone Extraction, Immunoprecipitation, Immunoblotting, and Acetylome Profiling

Histones were extracted from fresh mouse brain tissue or HT22 cells. Coomassie Blue staining confirmed histone enrichment (Figure S1C). Equal amounts of histone were subjected to H2B immunoprecipitation, followed by acetyl‐lysine immunoblotting to quantify pan‐H2Bac. Input, IgG, and H2B‐IP fractions served as workflow controls (Figure S1D). BCL2, BAX, and GALC were quantified by conventional immunoblotting. Antibodies are listed in Table S1.

Histone acetylome profiling was performed by Applied Protein Technology (Shanghai, China). Acetylated peptides were enriched using an Acetyl‐Lysine Motif kit and analyzed by liquid chromatography–tandem mass spectrometry on a Q Exactive instrument. Raw data were processed with MaxQuant [27]. Sequence conservation and nucleosome localization were assessed using public sequence data and PDB 3X1V [28]. Sample design and candidate‐site screening criteria are provided in the Supplementary Methods.

### 2.5 Brain Injury, Behavioral, Histological, and Ultrastructural Assessments

Neurological function was assessed before ICH and on days 1, 3, and 7 using the modified neurological severity score, wire‐hang, corner‐turn, and cylinder tests [29]. Hematoma volume was calculated from serial 1‐mm coronal sections [30], and ipsilateral brain water content was measured by the wet‐dry method. Investigators blinded to group allocation performed behavioral, histological, and imaging analyses.

Cell death was assessed by TUNEL staining in mouse brain sections and HT22 cells. Luxol fast blue (LFB), myelin basic protein (MBP), and neurofilament 200 (NF200) staining evaluated myelin and axonal structures in the predefined regions shown in figure 4A [31]. Transmission electron microscopy was used to assess axon‐myelin ultrastructure [32]. The G‐ratio was calculated as axon diameter divided by myelinated‐fiber diameter. Anatomical sampling, staining, and ultrastructural procedures are detailed in the Supplementary Methods.

### 2.6 RNA Sequencing, Targeted Metabolomics, CUT&Tag, and qRT‐PCR

RNA‐sequencing libraries were prepared by mRNA enrichment, cDNA synthesis, fragment selection, and PCR amplification, followed by Illumina NovaSeq 6000 sequencing and differential‐expression and pathway analyses. Targeted metabolomics was performed using ultrahigh‐performance liquid chromatography–tandem mass spectrometry. Sample sources, comparisons, and sample sizes are provided in the Supplementary Methods.

Total‐H2B CUT&Tag used an anti‐H2B antibody to guide pA‐Tn5 tagmentation of DNA adjacent to H2B [33]. Sequenced peaks were annotated relative to transcription start sites, and peak‐associated genes underwent Gene Ontology analysis. The *Galc* locus was visualized in the Integrative Genomics Viewer; experimental details are provided in the Supplementary Methods.

Quantitative reverse‐transcription PCR measured *Galc* transcripts in HT22 cells and inflammation‐related transcripts in mouse brain tissue. RNA was extracted and reverse‐transcribed before quantitative amplification. Primer sequences are listed in Table S2.

### 2.7 Statistical Analysis

Analyses were performed in GraphPad Prism 8.0.2, and data are presented as mean ± SD. Normality and variance were assessed before group comparisons. Student's *t*‐test or one‐way ANOVA with Tukey correction was used for normally distributed data with equal variances; Welch's test was used when variances differed. Mann–Whitney U or Kruskal–Wallis tests were used for nonnormal data, with multiplicity adjustment where appropriate. Repeated measures and axon‐level data were analyzed using mixed‐effects models. Exact sample sizes are reported in the figure legends. Two‐sided *p* < 0.05 was considered significant.

## 3 Results

### 3.1 ICH and Hb Exposure Reduce Pan‐H2Bac and Nominate H2BK109ac as a Candidate Site

We first measured pan‐H2Bac in mouse brain tissue after ICH. Pan‐H2Bac decreased rapidly, reached its lowest level on day 1, and recovered toward the uninjured control level on days 3 and 7 (Figure 1B; *n* = 6/group). Because neuronal and axonal injury directly contributes to neurological dysfunction after ICH, we next asked whether a major blood‐derived toxic component elicited a similar response in neuron‐like cells. Hb reduced pan‐H2Bac in HT22 cells, with the lowest signal at 12 h and partial recovery by 24 h (Figure 1C; *n* = 6/group). All Hb concentrations tested at 12 h also reduced pan‐H2Bac (Figure 1D; *n* = 6/group). We therefore identified reduced H2B acetylation as a reproducible response to hemorrhagic stress in brain tissue and HT22 cells.

**FIGURE 1:**
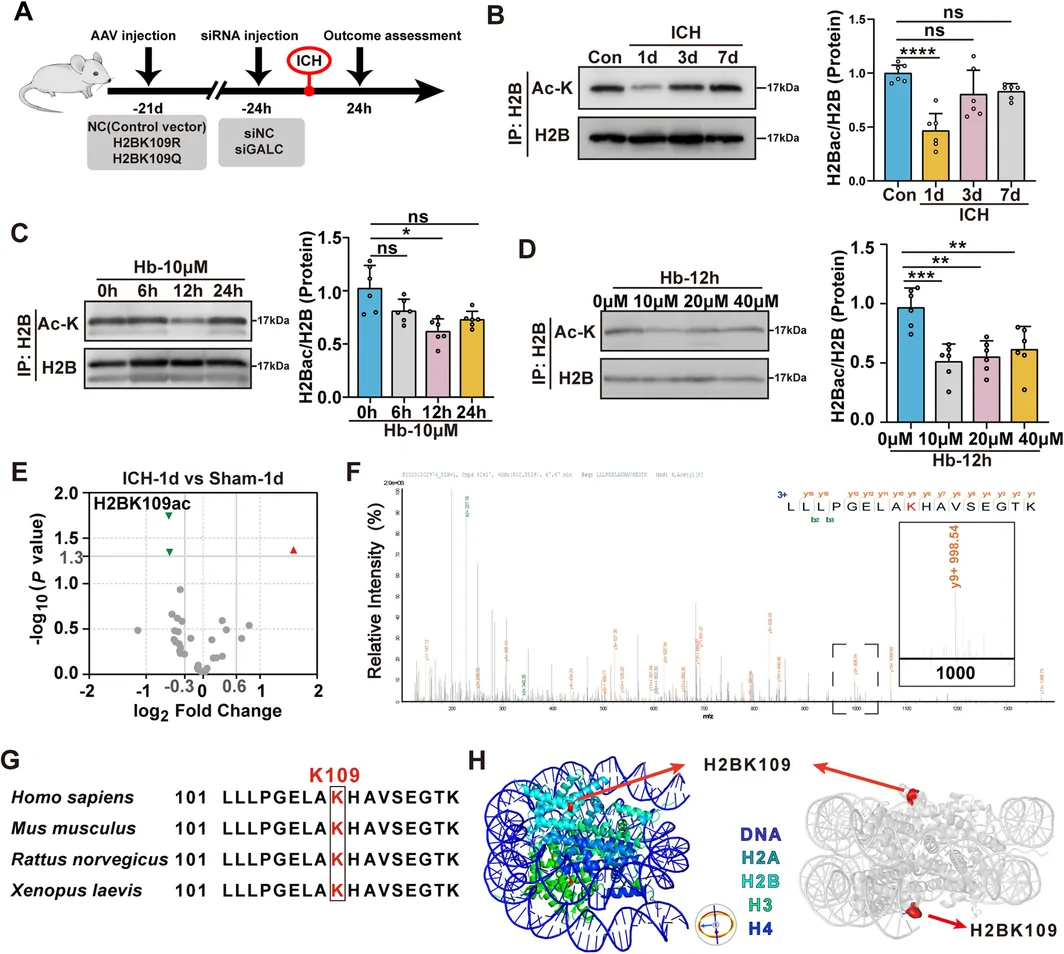
ICH and Hemoglobin (Hb) exposure reduce pan‐H2B acetylation and nominate H2BK109ac as an exploratory candidate. (A) Abbreviated timeline of AAV or siRNA delivery, ICH induction, and sampling at 24 h. (B) H2B immunoprecipitation followed by acetyl‐lysine immunoblotting in uninjured control (Con) brain tissue and brain tissue collected 1, 3, and 7 days after ICH (*n* = 6/group). (C) Pan‐H2Bac in HT22 cells exposed to 10 μmol/L Hb for 0–24 h and (D) to 0–40 μmol/L Hb for 12 h (*n* = 6/group). (E) Exploratory brain histone acetylome volcano plot comparing day‐1 ICH with day‐1 sham tissue (*n* = 3/group); the green triangle denotes H2BK109ac. (F) MS/MS spectrum localizing acetylation to H2BK109. (G) Cross‐species conservation of the H2BK109 region. (H) Location of H2BK109 in the nucleosome structure (PDB, 3X1V). Data are presented as mean ± SD. One‐way ANOVA with Tukey correction was used for B and D; Welch ANOVA with multiplicity‐adjusted between‐group comparisons was used for C. *p < 0.05, **p < 0.01, ***p < 0.001, ****p < 0.0001; ns, not significant.

Based on the in vivo temporal profile, we performed acetylome profiling of mouse brain tissue on day 1 after ICH. We identified 122 acetylation sites and quantified 64 of them (Figure S1E,F). H2BK109ac levels were lower after ICH than after sham surgery (fold change, 0.66; log2 fold change, −0.60; nominal *p* = 0.018; Figure 1E; *n* = 3/group), and the MS/MS spectrum localized the modification to K109 (Figure 1F). K109 was conserved across the species examined and located on the nucleosome surface (Figure 1G,H). We therefore selected H2BK109ac as a candidate site for functional testing.

### 3.2 H2BK109Q Reduces Acute Brain Injury and Improves Neurological Function

We next tested whether the modeled H2BK109 state altered the effects of acute ICH injury. After confirming local AAV expression in the mouse brain (Figure 2A), pan‐H2Bac levels were higher in H2BK109Q‐expressing brain tissue than in NC and H2BK109R tissue on day 1 after ICH (Figure 2B; *n* = 4/group). H2BK109Q‐expressing mice had smaller hematomas than NC mice on days 1, 3, and 7, whereas H2BK109R‐expressing mice did not show a comparable change (Figure 2C; *n* = 8/group). Brain water content was also lower in H2BK109Q‐expressing mice (Figure 2D; *n* = 6/group).

**FIGURE 2:**
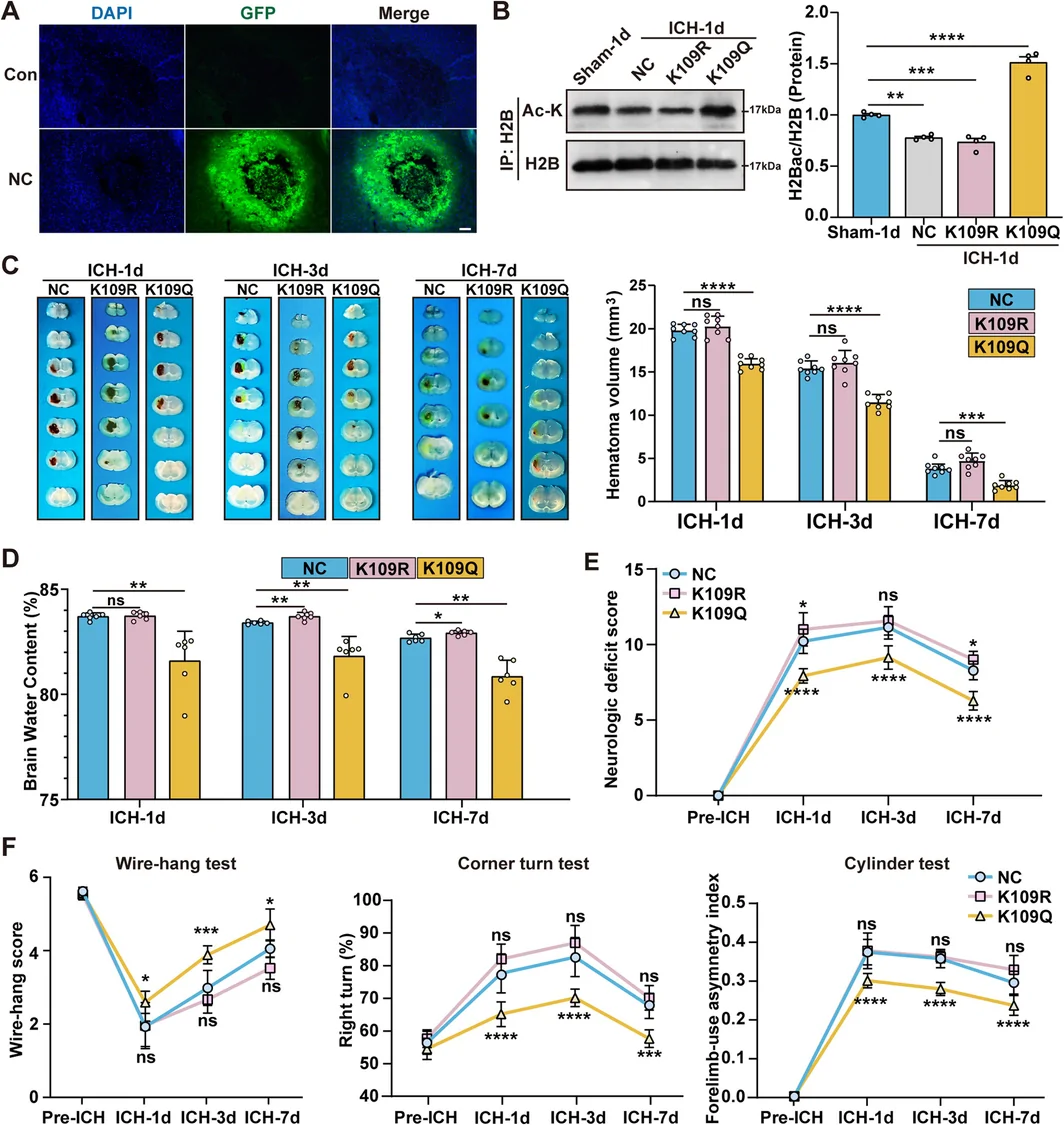
H2BK109Q reduces acute brain injury and improves neurological outcomes after ICH. (A) Regional ZsGreen signal in the brains of nontransduced controls (Con) and control‐vector (NC) mice (Scale bar = 100 μm). (B) Pan‐H2Bac in brain tissue from sham, NC, H2BK109R, and H2BK109Q mice on day 1 after ICH (*n* = 4/group). (C) Serial coronal sections and hematoma volume in independent terminal cohorts on days 1, 3, and 7 after ICH (*n* = 8/group/time point). (D) Ipsilateral brain water content (*n* = 6/group). (E) Modified neurological deficit score (*n* = 14/group). (F) Wire‐hang, corner‐turn, and cylinder tests (*n* = 8/group). Longitudinal behavioral data were analyzed using mixed‐effects models with mouse as a random intercept; other tests were selected based on the data distribution and variance, as described in Methods. Data are presented as mean ± SD. *p < 0.05, **p < 0.01, ***p < 0.001, ****p < 0.0001; ns, not significant.

These reductions in tissue injury were accompanied by improved neurological performance. H2BK109Q‐expressing mice had lower modified neurological severity scores throughout the follow‐up period (Figure 2E; *n* = 14/group), along with better wire‐hang performance, less lateralized turning, and greater spontaneous use of the affected forelimb (Figure 2F; *n* = 8/group). Several pro‐ and anti‐inflammatory transcripts were also lower in H2BK109Q‐expressing brain tissue on day 1 (Figure S2A,B), indicating broad attenuation of the acute inflammatory response rather than a shift toward a single polarized state.

### 3.3 H2BK109Q Limits Apoptosis in Vivo and Under Matched Hb Exposure

We next assessed apoptosis. In H2BK109Q‐expressing brain tissue, BCL2 increased, BAX decreased, and the BCL2/BAX ratio increased on days 1, 3, and 7 after ICH (Figure 3A; *n* = 6/group). H2BK109Q‐expressing mice also had less TUNEL‐positive perihematomal tissue on day 1 (Figure 3B; *n* = 8/group).

**FIGURE 3:**
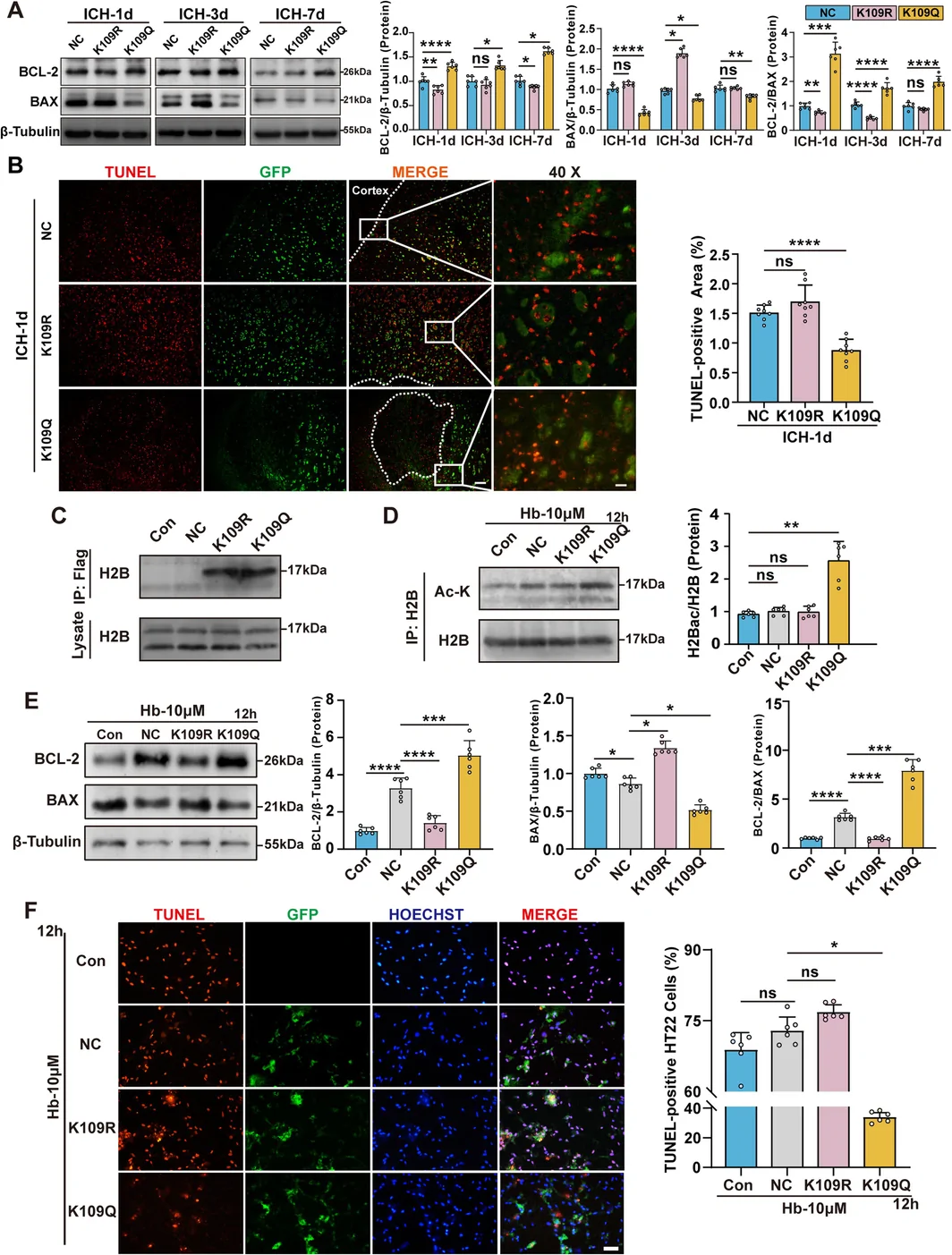
H2BK109Q reduces cell death in vivo and in HT22 cells treated with hemoglobin (Hb). (A) BCL2, BAX, and BCL2/BAX in ipsilateral brain tissue 1, 3, and 7 days after ICH (*n* = 6/group). (B) Perihematomal TUNEL staining on day 1 after ICH (*n* = 8/group; Scale bar = 100 μm; enlarged images, scale bar = 25 μm). White dotted lines delineate the cortex in NC (to the left of the line) and hematoma regions in K109R (below the line) and K109Q (within the outline). (C) Flag immunoprecipitation confirming expression of the H2BK109R and H2BK109Q constructs in HT22 cells. (D) Pan‐H2Bac, (E) BCL2 and BAX, and (F) TUNEL staining in Con, NC, H2BK109R, and H2BK109Q HT22 cells exposed to the same 10 μmol/L Hb challenge for 12 h (*n* = 6/group; F, Scale bar = 50 μm). Con cells were untransduced; NC cells carried the control vector. Data are presented as mean ± SD. Tests were selected based on normality and variance, as described in the Methods. *p < 0.05, **p < 0.01, ***p < 0.001, ****p < 0.0001; ns, not significant.

We then tested whether H2BK109Q altered HT22 cells' response to a fixed blood‐derived insult. Flag immunoprecipitation confirmed stable construct expression (Figure 3C). Under the same Hb challenge, H2BK109Q‐expressing cells had higher pan‐H2Bac, whereas NC and H2BK109R‐expressing cells remained close to the Con group (Figure 3D; *n* = 6/group). H2BK109Q‐expressing cells also showed the most favorable BCL2/BAX profile (Figure 3E; *n* = 6/group). Their TUNEL‐positive fraction was lower than that of H2BK109R‐expressing cells and numerically lower than those of Con and NC cells (Figure 3F; *n* = 6/group). Thus, the in vitro cell death‐related findings were directionally consistent with the in vivo phenotype under matched Hb exposure.

### 3.4 H2BK109Q Preserves Acute Axon‐Myelin Integrity

Because axonal and myelin injury contributes to neurological dysfunction after ICH, we examined the perihematomal CPu and the ipsilateral CC on day 1 (Figure 4A). H&E staining showed better‐preserved local morphology in H2BK109Q‐expressing tissue than in NC and H2BK109R tissue (Figure S3A). NC and H2BK109R sections contained more vacuolated and discontinuous LFB‐stained fibers, whereas H2BK109Q‐expressing mice retained larger LFB‐positive areas in both regions (Figure 4B,C; *n* = 8/group). MBP‐positive areas (Figure 4D,E; *n* = 7/group, *n* = 8/group) and NF200 signal (Figure 4F,G; *n* = 8/group) were also better preserved. Supplemental NF200/GFP images showed more complete NF200‐positive structures within H2BK109Q‐expressing regions (Figure S3B).

**FIGURE 4:**
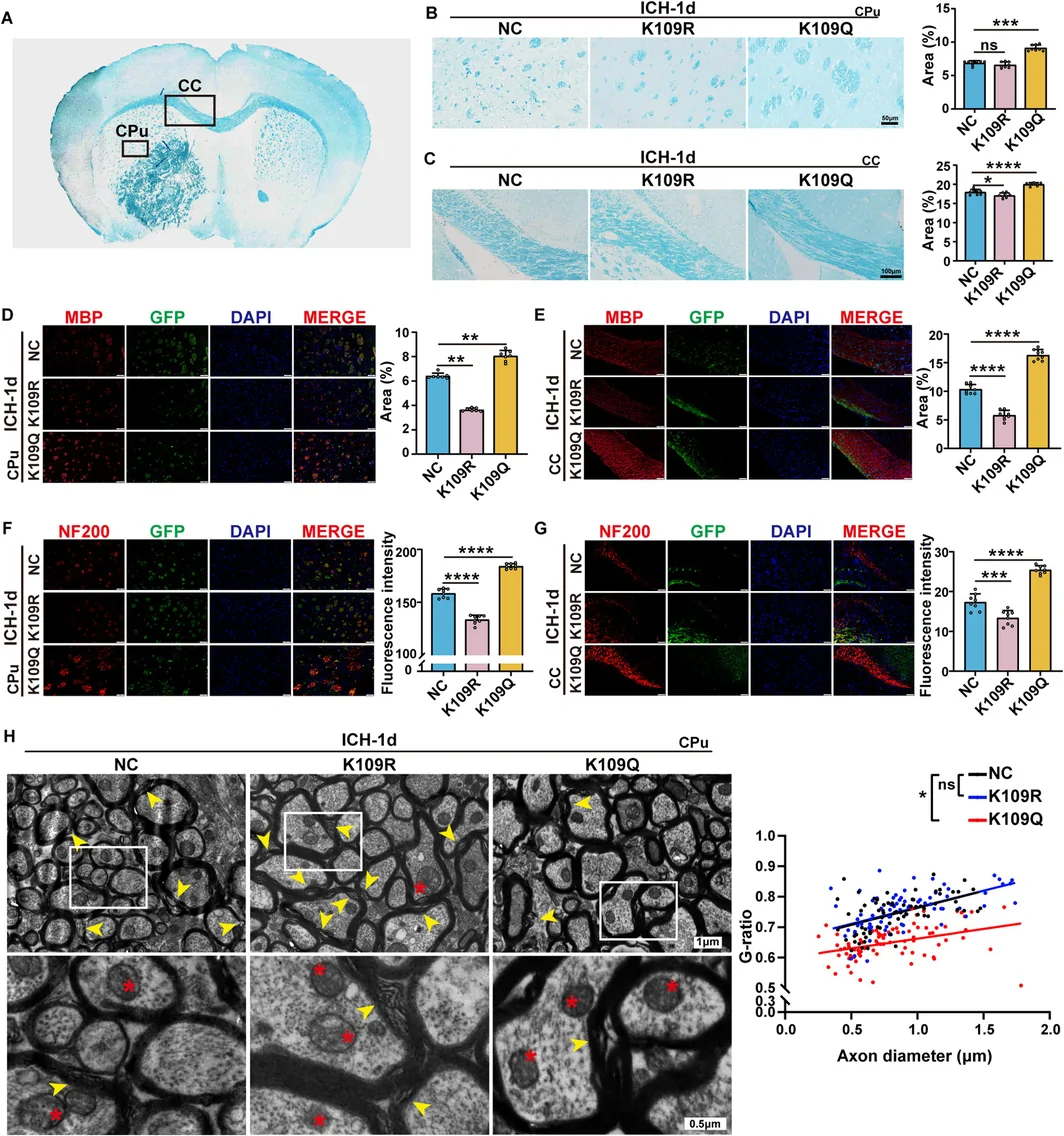
H2BK109Q preserves acute axon‐myelin integrity after intracerebral hemorrhage (ICH). (A) LFB‐stained coronal section shows the caudate putamen (CPu) and corpus callosum (CC) sampling regions. (B, C) LFB‐positive area in the CPu and CC (*n* = 8/group; B, Scale bar = 50 μm; C, Scale bar = 100 μm). (D, E) MBP‐positive area in the CPu (*n* = 7/group; Scale bar = 50 μm) and CC (*n* = 8/group; Scale bar = 50 μm). (F, G) NF200 signal intensity in the CPu and CC (*n* = 8/group; Scale bar = 50 μm). (H) CPu TEM images and axon diameter‐G‐ratio relationships (*n* = 3 mice/group; 74 NC, 79 H2BK109R, and 80 H2BK109Q axons; Scale bar = 1 μm; enlarged images, scale bar = 0.5 μm). Yellow arrows and red asterisks indicate representative myelin profiles and intra‐axonal organelles, respectively. Data are presented as mean ± SD. *p < 0.05, **p < 0.01, ***p < 0.001, ****p < 0.0001; ns, not significant.

TEM revealed loose or disrupted myelin and abnormal axonal organelles in NC and H2BK109R tissue, whereas H2BK109Q tissue retained more compact myelin and better‐preserved axonal ultrastructure (Figure 4H; *n* = 3/group). Per‐mouse mean G‐ratio did not differ among groups, but the G‐ratio–axon diameter model showed a group‐by‐diameter interaction (*p* = 0.0012). Slopes differed between NC and H2BK109Q (adjusted *p* = 0.0313) and between H2BK109R and H2BK109Q (adjusted *p* = 0.0007), but not between NC and H2BK109R. In the supplemental analysis, the overall myelin‐thickness comparison was significant (*p* = 0.0197), although no Holm‐adjusted pairwise comparison reached significance (Figure S3C). These day‐1 histological and ultrastructural findings indicate acute preservation of axon–myelin structure.

### 3.5 Molecular Analyses Prioritize GALC as a Candidate Effector

We first used RNA sequencing of mouse brain tissue to identify candidate effectors of the H2BK109Q‐associated protective phenotype. Compared with H2BK109R tissue, H2BK109Q tissue showed 338 upregulated and 767 downregulated genes (Figure 5A; *n* = 3/group). Gene‐set enrichment analysis highlighted neurotransmission‐related pathways, inositol phosphate metabolism, phosphatidylinositol signaling, and glycosphingolipid biosynthesis (Figure 5B). Complete KEGG and Gene Ontology results are shown in Figure S2C–E. These transcriptomic findings informed the subsequent metabolic analysis.

**FIGURE 5:**
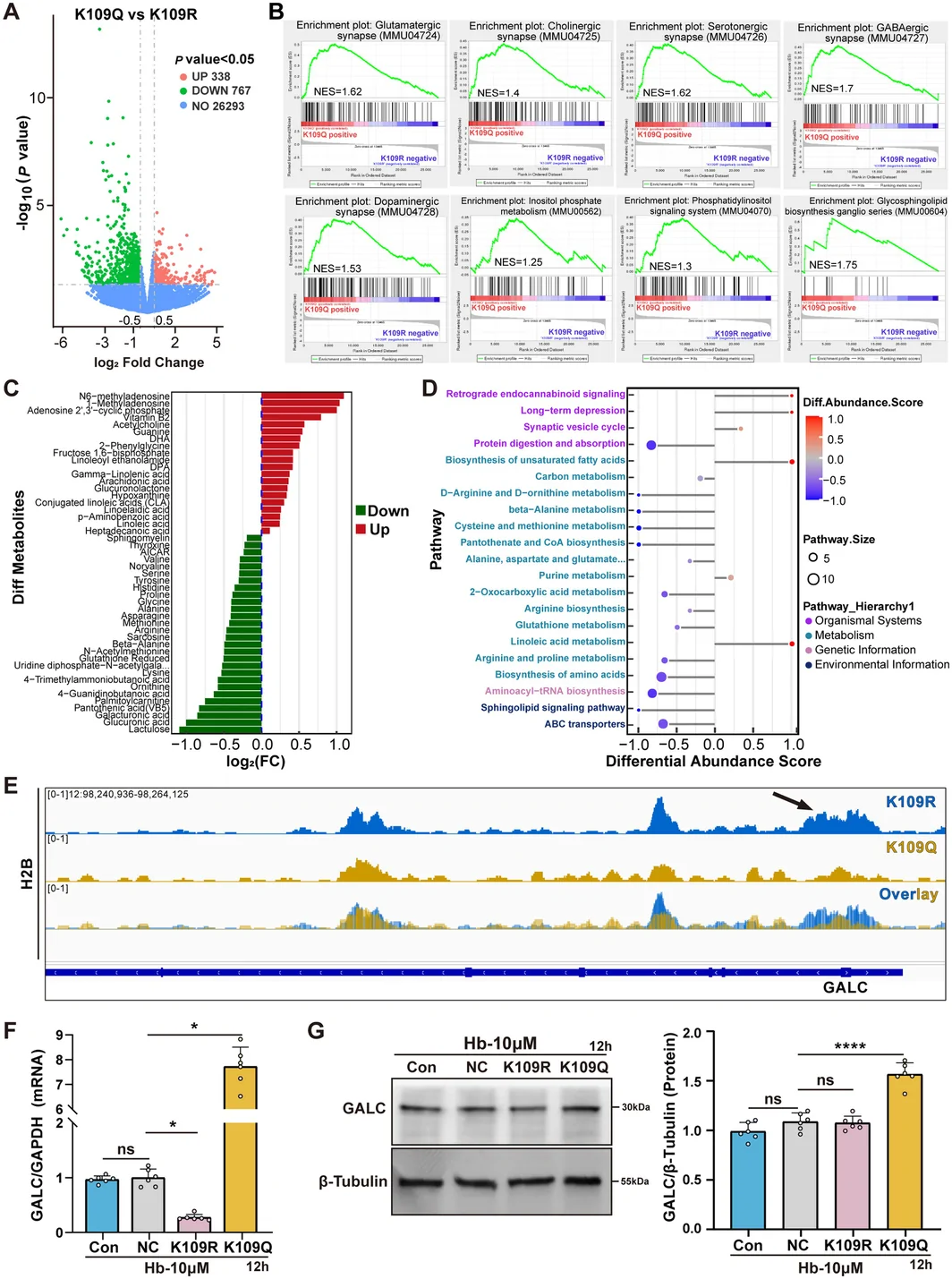
Transcriptomic, metabolomic, and chromatin analyses implicate lipid‐related pathways and prioritize GALC as a candidate effector of H2BK109Q‐associated protection. (A) Exploratory RNA‐seq volcano plot comparing H2BK109Q and H2BK109R brain tissue on day 1 (*n* = 3/group); at nominal *p* < 0.05, 338 genes were upregulated, and 767 were downregulated. (B) Representative gene‐set enrichment plots comparing H2BK109Q and H2BK109R brain tissue. (C) Differential metabolites and (D) differential pathway‐abundance scores in H2BK109Q and H2BK109R HT22 cells under the same Hb challenge (*n* = 3/group). (E) Total‐H2B CUT&Tag signal at the Galc locus (*n* = 1 per condition; descriptive profiles only). (F) Galc mRNA and (G) GALC protein in HT22 cells under the same Hb challenge (*n* = 6/group). Data are presented as mean ± SD. Tests were selected based on normality and variance, as described in the Methods. *p < 0.05, ****p < 0.0001; ns, not significant.

We next compared HT22 cells expressing H2BK109Q or H2BK109R under matched Hb exposure. Targeted metabolomics distinguished the two groups and identified differences in lipid, amino acid, nucleotide, and energy metabolites (Figure 5C and Figure S4A–E). Pathway analysis implicated unsaturated fatty acid biosynthesis, sphingolipid signaling, synaptic vesicle cycling, and membrane‐related metabolism (Figure 5D).

Given the convergence of glycosphingolipid‐ and galactolipid‐related pathways and the established relevance of GALC to axon–myelin homeostasis, we selected GALC for subsequent validation. Total‐H2B CUT&Tag identified 24,781 shared peaks, with 9512 and 6285 peaks specific to H2BK109R and H2BK109Q cells, respectively; global heatmaps summarized H2B distribution across gene regions (Figure S4F). At the *Galc* locus, cells expressing H2BK109Q showed a reduced total‐H2B signal near the promoter and transcription start region compared to those expressing H2BK109R (Figure 5E). Following the same Hb challenge, H2BK109Q‐expressing cells demonstrated elevated levels of both *Galc* mRNA and GALC protein (Figure 5F,G; *n* = 6/group). These findings highlight GALC as a potential effector implicated in the protective effects associated with H2BK109Q.

### 3.6 Galc Knockdown Weakens H2BK109Q‐Associated Preservation of Axon‐Myelin in the CPu and CC

We locally delivered siGALC to H2BK109Q‐expressing mice to assess its role in axon–myelin protection. Immunoblotting confirmed lower GALC protein in the injected brain region (Figure 6A; *n* = 6/group). Untreated H2BK109Q and K109Q‐siNC mice did not differ across the six histological measures. K109Q‐siGALC mice, however, had reduced LFB‐positive areas in the CPu and CC (Figure 6B,C; *n* = 6/group), smaller MBP‐positive areas (Figure 6D,E; *n* = 6/group), and lower NF200 signals (Figure 6F,G; *n* = 6/group) than both comparison groups. These findings indicate that Galc knockdown weakens the protective effects of H2BK109Q against white matter injury.

**FIGURE 6:**
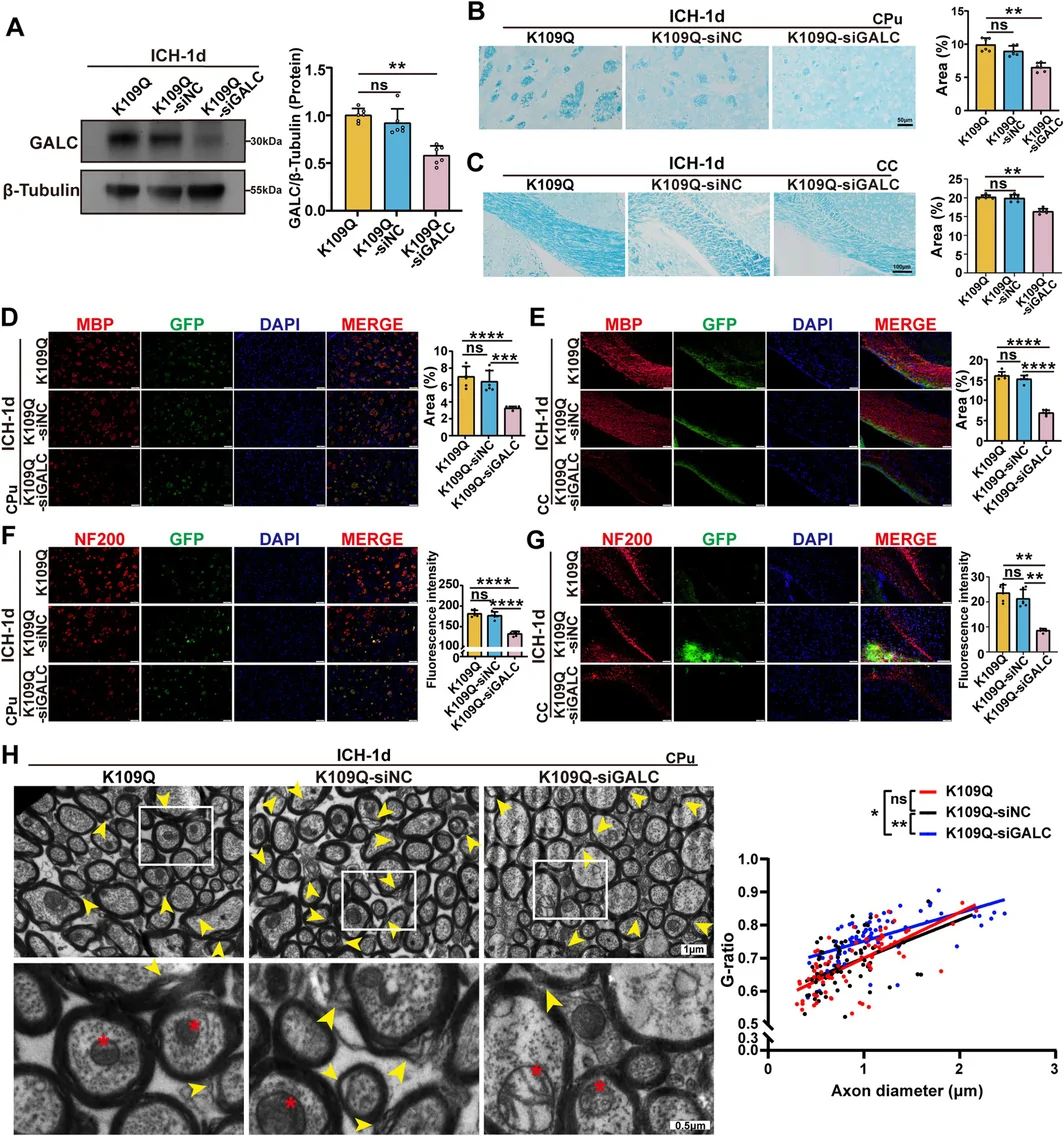
Galc knockdown attenuates H2BK109Q‐mediated axon‐myelin protection in the CPu and CC. (A) GALC immunoblotting after regional delivery of siNC or siGALC in H2BK109Q mice. (B, C) LFB‐positive area in the CPu and CC (B, Scale bar = 50 μm; C, Scale bar = 100 μm). (D, E) MBP‐positive area in the CPu and CC (Scale bar = 50 μm). (F, G) NF200 signal in the CPu and CC (Scale bar = 50 μm). For A‐G, *n* = 6/group. (H) CPu TEM images and axon diameter‐G‐ratio plots (*n* = 3 mice/group; 68 H2BK109Q, 71 K109Q‐siNC, and 68 K109Q‐siGALC axons; Scale bar = 1 μm; enlarged images, scale bar = 0.5 μm). Yellow arrows and red asterisks indicate representative myelin profiles and intra‐axonal organelles, respectively. Data are presented as mean ± SD. *p < 0.05, **p < 0.01, ***p < 0.001, ****p < 0.0001; ns, not significant.

TEM supported the histological findings. K109Q‐siGALC brain tissue showed greater myelin irregularity and separation, along with axonal mitochondrial abnormalities (Figure 6H; *n* = 3/group). Per‐mouse mean G‐ratio and mean myelin thickness did not differ among groups. However, the myelin thickness–axon diameter model showed a group‐by‐diameter interaction (*p* = 0.0018). Slopes did not differ between untreated H2BK109Q and K109Q‐siNC mice (adjusted *p* = 0.3781), but differed between H2BK109Q and K109Q‐siGALC (adjusted *p* = 0.0206) and between K109Q‐siNC and K109Q‐siGALC mice (adjusted *p* = 0.0023; Figure S3D). Together with Figure 6B–G, these data show that GALC contributes to H2BK109Q‐associated axon–myelin preservation.

## 4 Discussion

This study identifies H2BK109 as a functionally relevant residue in acute ICH injury. ICH reduced pan‐H2Bac in mouse brain tissue, and Hb produced a similar response in HT22 cells; exploratory acetylome profiling also identified reduced endogenous H2BK109ac. H2BK109Q‐expressing mice showed smaller hematomas, less edema and apoptosis, better neurological function, and greater preservation of axon–myelin structure. HT22 cells exposed to the same Hb challenge reproduced the survival‐associated effect, and regional *Galc* knockdown weakened structural protection in H2BK109Q‐expressing mice. These findings link the modeled H2BK109 state to acute brain injury and identify GALC as a contributor to the axon–myelin phenotype.

Most epigenetic studies of ICH have examined H3/H4 acetylation, histone lactylation, or broad HDAC inhibition [12, 13, 14, 15, 16, 17]. Because broad enzyme inhibition affects many histone and nonhistone substrates, these studies cannot attribute protection to a specific residue. H2B acetylation marks active enhancers, can persist independently of ongoing transcription, and participates in apoptosis‐associated chromatin remodeling [18, 19, 20]. In our acetylome screen, H2BK109ac was reduced after ICH and localized by peptide fragmentation. K109 is conserved and located on the nucleosome surface, but these features alone do not establish function. The subsequent K109Q/K109R comparison provides functional evidence that the modeled K109 state influences ICH injury.

In mouse brain tissue and HT22 cells, H2BK109Q was associated with higher pan‐H2Bac levels. In HT22 cells, H2BK109Q was also associated with a lower total H2B signal near Galc, increased Galc mRNA, and higher GALC protein levels. These findings suggest that H2BK109Q alters H2B‐associated chromatin organization at *Galc*. Total‐H2B CUT&Tag cannot distinguish nucleosome disassembly, H2A–H2B exchange, or other forms of chromatin reorganization, so H2BK109ac‐specific measurements will be required to test this relationship at the endogenous site.

At the tissue level, H2BK109Q preserved LFB, MBP, NF200, and corpus callosum structure. These findings align with single‐cell, histopathological, and clinical evidence showing coordinated responses of neurons, oligodendroglia, microglia, monocytes/macrophages, and other infiltrating immune cells after ICH [8, 34, 35, 36, 37, 38, 39, 40, 41]. Complement signaling, T‐cell checkpoint regulation, gut‐brain immune trafficking, and IL‐10 delivery have also emerged as potentially modifiable components of the post‐ICH immune response [42, 43, 44, 45, 46]. Recent bibliometric and bioinformatic studies further underscore the growing interest in the cellular and molecular heterogeneity of neuroinflammation after ICH [47, 48]. Lipid‐related signals in our transcriptomic and metabolomic data provide additional context because myelin integrity depends on tightly regulated lipid homeostasis, which is disrupted after ICH [49]. All structural measurements were obtained on day 1; the findings indicate reduced acute axonal and myelin degeneration rather than remyelination or regeneration.

Smaller hematomas are an important component of the H2BK109Q protective phenotype and may reduce subsequent exposure to blood‐derived injury [50, 51, 52]. Under a fixed Hb challenge, H2BK109Q‐expressing HT22 cells also showed a higher BCL2/BAX ratio and lower TUNEL positivity than H2BK109R‐expressing cells. The cell experiments therefore support an additional survival‐associated effect under controlled exposure without establishing a direct cell‐autonomous effect on the in vivo axon–myelin unit.

GALC was selected because glycosphingolipid‐ and galactolipid‐related pathways converged across the molecular analyses and because GALC has established roles in neural lipid metabolism. Neuron‐specific loss of *Galc* causes axonal degeneration, whereas GALC in oligodendrocytes and microglia contributes to myelin homeostasis [53, 54]. Human myelinating organoids and therapeutic models also show GALC activity across neural cell populations [55, 56]. In our experiments, siGALC reduced LFB, MBP, and NF200 measures in both CPu and CC relative to untreated and siNC‐treated H2BK109Q mice. These loss‐of‐function data identify GALC as a functional contributor to H2BK109Q‐associated axon–myelin protection rather than merely an omics‐derived candidate.

TEM corroborated the regional histological findings but showed that the effect was not uniform across all fibers. Mean G‐ratio and mean myelin thickness did not differ at the mouse level, whereas the myelin thickness–axon diameter relationship changed after siGALC. This caliber‐dependent pattern was consistent with reduced LFB and MBP measures and with qualitative ultrastructural abnormalities. GALC is therefore one contributor to the H2BK109Q phenotype, while changes in hematoma volume, cell death, and inflammatory transcripts suggest that additional H2BK109‐responsive pathways may also participate.

Several aspects of the study define the next experimental steps. K109Q and K109R are structural substitutions rather than endogenous post‐translational states; AAV was delivered before ICH, and only young male mice were studied. The omics analyses were exploratory, whereas total‐H2B CUT&Tag provided a single descriptive profile per condition rather than an H2BK109ac‐specific measurement. Future studies should establish a validated H2BK109ac‐specific assay, determine its temporal and cell‐type distribution, and test post‐injury manipulation of endogenous H2BK109 and GALC.

In summary, H2BK109Q‐expressing mice exhibited less acute brain injury and better‐preserved axon–myelin structure after ICH. H2BK109Q‐expressing HT22 cells also resisted a matched Hb challenge, and regional *Galc* knockdown reduced axon–myelin preservation. These findings identify H2BK109 as a residue of interest in ICH and GALC as a contributor to the protective phenotype.

## Author Contributions

Sinan Jin designed the study, analyzed and interpreted the data, prepared the figures, and drafted the manuscript. Meng Yang and Jincheng Meng performed part of the in vitro experiments and contributed to data collection. Ziyi Han and Pengtian Lu contributed to data collection. Yousef Rastegar‐Kashkooli and Junmin Wang contributed to data analysis and experimental design, respectively. Jian Wang provided scientific guidance and revised the manuscript. He Wu supervised the study, obtained funding, and revised the manuscript. All authors reviewed and approved the final manuscript.

## Funding

This work was supported by the National Natural Science Foundation of China (81671142) and the Natural Science Foundation for Distinguished Young Scholars of Heilongjiang Province, China (JC2018022).

## Ethics Statement

All animal procedures were approved by the Ethics Review Committee for the Use and Welfare of Experimental Animals of the First Affiliated Hospital of Harbin Medical University, China (IACUC No. 2022132), and were conducted and reported in accordance with ARRIVE 2.0.

## Conflicts of Interest

Jian Wang is the Associate Editor of *CNS Neuroscience and Therapeutics* and a co‐author of this article. He was excluded from editorial decision‐making related to the acceptance and publication of this article. Editorial decision‐making was handled independently by the other editor to minimize bias. The other authors declare no conflicts of interest.

## Supporting information


**Table S1:** Antibodies used in this study.
**Table S2:** Primer sequences used in this study.
**Figure S1:** Experimental localization and acetylome quality‐control information.
(A) Stereotaxic surface coordinates. (B) Injection depth and the hematoma, perihematomal, CPu, and CC analysis regions. (C) Coomassie‐stained, histone‐enriched brain extracts used for acetylome profiling (day‐1 sham and day‐1 ICH; *n* = 3/group). (D) Validation of the H2B immunoprecipitation/acetyl‐lysine immunoblotting workflow using input, IgG, and H2B‐IP fractions. (E) Numbers of identified and quantified acetylated proteins, peptides, and sites. (F) Heat map of quantified acetylation sites, with H2BK109ac indicated.
**Figure S2:** Inflammation‐related transcripts and complete RNA‐seq enrichment results.
(A) Tnf, Il6, Il1b, Cd86, and Nlrp3 transcripts. (B) Tgfb, Il4, Il10, and Arg1 transcripts in NC, H2BK109R, and H2BK109Q brain tissue on day 1 (*n* = 6/group). (C) KEGG enrichment of mRNAs significantly upregulated in the brains of H2BK109Q mice compared with H2BK109R mice, and (D) Gene Ontology biological process, cellular component, and molecular function results for these significantly upregulated mRNAs. (E) Detailed enrichment maps of these significantly upregulated mRNAs in glutamatergic, cholinergic, serotonergic, and GABAergic synapse pathways. Data are presented as mean ± SD. *p < 0.05, **p < 0.01, ***p < 0.001, ****p < 0.0001.
**Figure S3:** Additional histological and TEM measurements.
(A) H&E‐stained whole coronal sections and enlarged CC and CPu regions from Con, NC, H2BK109R, and H2BK109Q mice (Scale bar = 625 μm; enlarged images, scale bar = 100 μm). (B) Additional NF200/GFP images in NC, H2BK109R, and H2BK109Q tissue (Scale bar = 20 μm; enlarged images, scale bar = 10 μm). (C) TEM‐derived myelin thickness in NC, H2BK109R, and H2BK109Q mice (*n* = 3 mice/group). (D) Myelin thickness after siNC or siGALC delivery in H2BK109Q mice (*n* = 3 mice/group; 72 H2BK109Q, 74 K109Q‐siNC, and 73 K109Q‐siGALC axons). Symbols above D denote Holm‐adjusted comparisons of mixed‐effects‐model slopes for myelin thickness versus axon diameter: H2BK109Q versus K109Q‐siNC, *p* = 0.3781; H2BK109Q versus K109Q‐siGALC, *p* = 0.0206; K109Q‐siNC versus K109Q‐siGALC, *p* = 0.0023. Axons were nested within mice and were not treated as independent biological replicates. *p < 0.05, **p < 0.01; ns, not significant.
**Figure S4:** Supplementary metabolomic and total‐H2B CUT&Tag results.
(A–E) Principal component analysis, volcano plot, metabolite heat map, KEGG summary, and sphingolipid pathway map for H2BK109Q and H2BK109R HT22 cells under the same Hb challenge (*n* = 3/group). (F) CUT&Tag peak Venn diagram and global total‐H2B signal.

## Data Availability

The data that support the findings of this study are available from the corresponding author upon reasonable request.
