# Supplementary material for: Acetylation‐like H2BK109Q attenuates acute brain injury and preserves axon‐myelin integrity after intracerebral hemorrhage
Source: CNS Neurosci Ther. 2026 Oct 9;32(10):e71172. doi: 10.1002/cns.71172 (PMC13650523; doi:10.1002/cns.71172)
Supplement: Supplementary file 1 [file CNS-32-e71172-s001.docx]

**Supporting Information**

**Acetylation-Like H2BK109Q Attenuates Acute Brain Injury and Preserves Axon-Myelin Integrity After Intracerebral Hemorrhage**

# Supplementary Methods

## Animals and experimental intracerebral hemorrhage

Male C57BL/6 mice (8 weeks old; approximately 22 g; Changsheng Biotechnology, Liaoning, China) were housed at 22-24 °C and 45%-65% relative humidity under a 12 h light/dark cycle with free access to food and water. Mice were allocated to experimental groups using a random-number table, and group assignments were managed using coded cage cards. No formal priori power calculation was performed. Following standardization of the collagenase ICH procedure in preliminary work, group sizes were determined with reference to previous experimental ICH studies and the anticipated variability of each endpoint; exact biological sample sizes are reported in the corresponding figure legends. No animals died or were excluded after randomization, no technical failures resulted in missing samples, and no outcome data were omitted. Only male mice were used to reduce variation associated with estrogen-related neuroprotection.^1^ All animal procedures were approved by the Ethics Review Committee for the Use and Welfare of Experimental Animals of the First Affiliated Hospital of Harbin Medical University (IACUC No. 2022132) and were conducted and reported in accordance with ARRIVE 2.0.^2^

ICH was induced by stereotaxic injection of collagenase VII-S.^3,4^ Mice were anesthetized with isoflurane (R510-22, RWD, China) and placed in a stereotaxic frame. Bregma was defined as the intersection of the sagittal and coronal sutures, and a burr hole was drilled 1.0 mm anterior and 2.2 mm left lateral to bregma (Figure S1A). The microsyringe needle was lowered to 2.7 mm below the skull surface and retained for 5 min, then raised to 2.6 mm below the skull surface for infusion. Collagenase VII-S (C0773, Sigma-Aldrich; 0.075 U/μL; 0.4 μL; total dose, 0.03 U per mouse) was infused into the left striatum at 0.2 μL/min over 2 min. Sham mice received 0.4 μL of saline at the same coordinates. After infusion, the needle was retained for 10 min, withdrawn to 2.0 mm below the skull surface, held for 5 min, and slowly removed (Figure S1B). At the designated time points, mice were deeply anesthetized with isoflurane, and brain tissue was collected.

## AAV and lentiviral vectors

Hanbio Tech (Shanghai, China) prepared all viral vectors. The lysine-109 codon of mouse *H2bc18* (AAA) was replaced with CAA for H2BK109Q or CGT for H2BK109R. For mouse experiments, AAV vectors were constructed with the pHBAAV-CMV-MCS-T2A-ZsGreen backbone. The AAV2/9 preparations were HBAAV2/9-m-H2bc18(K109Q)-3×Flag-ZsGreen (1.5 × 10^12^ vg/mL), HBAAV2/9-m-H2bc18(K109R)-3×Flag-ZsGreen (1.5 × 10^12^ vg/mL), and HBAAV2/9-ZsGreen control (1.8 × 10^12^ vg/mL).

For HT22 cells, lentiviral vectors were constructed with the pHBLV-CMV-MCS-3Flag-EF1-ZsGreen-T2A-PURO backbone. The mouse *H2bc18*(K109Q) or *H2bc18*(K109R) sequence was fused to a 3×Flag tag; ZsGreen and puromycin resistance supported transduction monitoring and stable selection. Lentiviral particles were packaged in 293T cells with the shuttle plasmid, psPAX2, and pMD2G using Lipofiter transfection reagent. Supernatants collected 48 and 72 h after transfection were concentrated by ultracentrifugation, and titers were determined by dilution counting in 293T cells. The preparations were HBLV-m-H2bc18(K109Q)-3×Flag-ZsGreen-PURO (3 × 10^8^ TU/mL), HBLV-m-H2bc18(K109R)-3×Flag-ZsGreen-PURO (2 × 10^8^ TU/mL), and HBLV-ZsGreen-PURO control (3 × 10^8^ TU/mL). Construct identity was confirmed by sequencing; sterility and mycoplasma testing were included in viral quality control.

## Intracerebral delivery of AAV and siRNA

AAV was delivered stereotactically 21 days before ICH. Investigators performing stereotaxic AAV delivery received only coded viral preparations and were unaware of construct identity. The injection site was the same ipsilateral striatal region used for the subsequent collagenase injection: 1.0 mm anterior, 2.2 mm lateral to the left, and 2.6 mm below the skull surface. A total volume of 2 μL was delivered via a microsyringe.

For GALC loss-of-function experiments, 100 pmol of negative-control siRNA (siNC) or GALC-specific siRNA (siGALC; RiboBio, Guangzhou, China) in RNase-free water (total volume, 2 μL) was injected into the same ipsilateral striatal/perihematomal region 24 h before ICH. No transfection reagent was used. The siGALC sense sequence was CUCAACCAACGACCUAUUATT and the antisense sequence was UAAUAGGUCGUUGGUUGAGTT. The siNC sense sequence was UUCUCCGAACGUGUCACGUTT and the antisense sequence was ACGUGACACGUUCGGAGAATT.

## HT22 cell culture, stable transduction, and Hb exposure

Mouse hippocampal neuron-like HT22 cells were obtained from iCell Bioscience (Shanghai, China) and maintained in Dulbecco's Modified Eagle Medium supplemented with 10% fetal bovine serum (A3160901, GIBCO), 100 μg/mL streptomycin, and 100 U/mL penicillin at 37 °C in 5% CO2. Stable ZsGreen control, H2BK109R, and H2BK109Q populations were generated by lentiviral transduction at an optimized multiplicity of infection of 90 using a half-volume, low-volume infection protocol, followed by selection with 2 μg/mL puromycin.

Hb exposure was used to model controlled blood-derived neuronal injury.^5,6^ Time-response experiments used 10 μmol/L Hb for 0-24 h, and concentration-response experiments used 0-40 μmol/L Hb for 12 h. Subsequent comparisons used 10 μmol/L Hb for 12 h. Con cells were untransduced HT22 cells; NC cells carried the ZsGreen control vector without an H2B insert. All comparison groups received the same Hb exposure.

## Histone extraction

Histones were extracted from fresh mouse brain tissue or HT22 cells with the Histone Extraction Kit (ab113476, Abcam) according to the manufacturer's instructions. Equal amounts of extracted protein were separated by SDS-PAGE and stained with Coomassie Blue (P0017FFT, Beyotime) for 30 min. Gels were washed with ultrapure water until discrete histone-enriched bands were visible, scanned, and used as quality-control evidence before immunoprecipitation or acetylome profiling (Figure S1D).

## Immunoprecipitation and immunoblotting

Histone immunoprecipitation used the Abcam immunoprecipitation kit (ab206996). Equal histone amounts were used for each immunoprecipitation, with an aliquot retained as input. Histone samples were incubated with anti-H2B, anti-Flag, anti-acetyl-lysine, or control IgG as required. H2B immunoprecipitation followed by acetyl-lysine immunoblotting measured pan-H2Bac; input, IgG, and H2B-IP fractions served as workflow controls (Figure S1C). Flag immunoprecipitation verified expression of the H2BK109 constructs in HT22 cells. BCL2, BAX, and GALC were measured by conventional immunoblotting. Antibody suppliers and catalog numbers are listed in Table S1.

## Histone acetylome profiling

Applied Protein Technology (Shanghai, China) performed histone lysine-acetylation profiling. Day-1 sham and day-1 ICH mouse brain samples were analyzed (n=3/group). Acetylated histone peptides were enriched with a PTMScan Acetyl-Lysine Motif kit (Cell Signaling Technology) and analyzed by liquid chromatography-tandem mass spectrometry on a Thermo Fisher Q Exactive mass spectrometer. Raw data were processed with MaxQuant.^7^ Candidate sites were screened using P<0.05 together with fold change <0.8 or >1.5. Candidate interpretation also considered MS/MS localization and subsequent functional validation. Sequence conservation was assessed across species, and the nucleosome location of H2BK109 was visualized using PDB 3X1V.^8^

## Brain injury and neurological assessments

For hematoma volume quantification, deeply anesthetized mice were euthanized, and brains were sectioned into serial 1-mm coronal slices. Sections were photographed with a grid scale, and hematoma area was measured by an investigator blinded to group allocation using ImageJ. Area was multiplied by section thickness and summed to obtain hematoma volume.^9^ For brain water content, fresh ipsilateral brain tissue was weighed, dried at 98 °C to constant mass, and reweighed. Water content was calculated as (wet weight - dry weight)/wet weight × 100%.

Neurological function was assessed before ICH and on days 1, 3, and 7. The modified neurological severity score evaluated global deficits; wire-hang, corner-turn, and cylinder tests assessed motor endurance and coordination, sensorimotor asymmetry, and spontaneous use of the affected forelimb, respectively.^10^ Investigators conducting behavioral testing were blinded to group allocation.

## Histology, immunofluorescence, and TUNEL staining

TUNEL staining was performed on frozen mouse brain sections and HT22 cells using a commercial kit (C1090, Beyotime). For mouse tissue analyses, the perihematomal caudate putamen (CPu) and the ipsilateral corpus callosum (CC) were sampled as shown in Figure 4A. Frozen sections were cut at 8 μm. For LFB staining, sections were dehydrated in ethanol, incubated overnight at 56 °C in 0.1% LFB solution, differentiated with lithium carbonate, counterstained with cresyl violet for 30 min, dehydrated through graded ethanol, cleared twice in xylene for 5 min, and then imaged.^11^

For immunofluorescence, 8-μm frozen sections were incubated with antibodies against MBP or NF200 (Table S1), then imaged under identical acquisition conditions within each experiment, and quantified in the predefined CPu and CC regions. H&E staining was used for general morphological assessment. Investigators were blinded to group allocation during image acquisition and quantitative analysis.

## Transmission electron microscopy

Ipsilateral CPu tissue blocks (approximately 1 mm thick) were fixed in 2% glutaraldehyde for 24 h, postfixed for 2 h in acetone containing osmium tetroxide, and embedded in epoxy resin. Ultrathin 80-nm sections were placed on 200-mesh grids, stained with uranyl acetate and lead citrate, and examined by transmission electron microscopy.^12^ G-ratio was calculated as axon diameter divided by the total diameter of the myelinated fiber. Myelin thickness was calculated from the axon and myelinated-fiber diameters. Mouse-level mean G-ratio and myelin thickness were the primary biological outcomes; axon-level measurements were nested within mice for diameter-dependent analyses.

## RNA sequencing

RNA sequencing compared day-1 brain tissue from H2BK109Q- and H2BK109R-expressing mice (n=3/group). Poly(A) mRNA was enriched from total RNA with poly-T oligo-attached magnetic beads. cDNA was synthesized, fragments of 370-420 bp were selected with AMPure XP beads (Beckman Colter), and amplified libraries were purified, pooled, and sequenced on an Illumina NovaSeq 6000 platform. Differential-expression, Gene Ontology, Kyoto Encyclopedia of Genes and Genomes, and gene set enrichment analyses were performed.

## Targeted metabolomics

Targeted metabolomics compared H2BK109Q- and H2BK109R-expressing HT22 cells after the same 10 μmol/L Hb exposure for 12 h (n=3 independent samples/group). Shanghai Applied Protein Technology performed the analysis using an AB Sciex QTRAP 6500+ mass spectrometer coupled to an Agilent 1290 Infinity UHPLC system. Metabolites were separated on Waters UPLC BEH C18 (2.1 × 100 mm, 1.7 μm) and Waters UPLC BEH Amide (2.1 × 100 mm, 1.7 μm) columns. Metabolite differences and pathway-abundance scores were used for pathway analysis.

## Total-H2B CUT&Tag

Total-H2B CUT&Tag compared H2BK109Q- and H2BK109R-expressing HT22 cells after matched Hb exposure. One descriptive library was obtained for each condition. Cell viability was checked with LUNA-FL technology. Cells were immobilized, and anti-H2B (ab1790, Abcam) guided pA-Tn5-mediated tagmentation of H2B-adjacent DNA.^13^ Libraries were amplified and sequenced, peaks were annotated relative to transcription start sites, and peak-associated genes underwent Gene Ontology analysis. Total-H2B signal at the *Galc* locus was visualized with Integrative Genomics Viewer.

## Quantitative reverse-transcription PCR

Total RNA was extracted using TIANGEN kits (DP431 and DP430). RNA concentration and purity were assessed before reverse transcription with the TOYOBO FSQ-201 kit. Quantitative PCR was performed on an ABI StepOne Real-Time PCR system in 20-μL reactions with the TOYOBO QPS-201 reagent. Primer sequences are listed in Table S2.

## Statistical analysis

Quantitative data are presented as mean ± SD. Shapiro-Wilk tests assessed normality, and Brown-Forsythe or Levene tests assessed homogeneity of variance as appropriate. Normally distributed data with equal variances were analyzed by unpaired two-tailed Student's t test or one-way ANOVA followed by Tukey multiple comparisons. Normally distributed data with unequal variances were analyzed by Welch tests with Holm-adjusted pairwise comparisons. Nonnormal data were analyzed by Mann-Whitney U or Kruskal-Wallis tests with Holm adjustment where appropriate.

Longitudinal outcomes were analyzed with mixed-effects models that included mouse as a random intercept. For TEM, mouse-level means were used for mean G-ratio and myelin-thickness comparisons. Axon-level measurements were nested within mice when modeling the relationships between axon diameter and G-ratio or myelin thickness. Tests were two-sided, and adjusted P<0.05 was considered statistically significant. Exact biological sample sizes are reported in the corresponding figure legends.

# Supplementary References

1. Koduri S, Keep RF, Hua Y, Chaudhary N, Pandey AS, Xi G. The two faces of estrogen in experimental hemorrhagic stroke. Transl Stroke Res. 2022;13(3):362-363. doi:10.1007/s12975-021-00942-0.

2. Percie du Sert N, Hurst V, Ahluwalia A, Alam S, Avey MT, Baker M, et al. The ARRIVE guidelines 2.0: updated guidelines for reporting animal research. PLoS Biol. 2020;18(7):e3000410. doi:10.1371/journal.pbio.3000410.

3. Jia P, He J, Li Z, Wang J, Jia L, Hao R, et al. Profiling of blood-brain barrier disruption in mouse intracerebral hemorrhage models: collagenase injection vs. autologous arterial whole blood infusion. Front Cell Neurosci. 2021;15:699736. doi:10.3389/fncel.2021.699736.

4. Wu H, Wu T, Xu X, Wang J, Wang J. Iron toxicity in mice with collagenase-induced intracerebral hemorrhage. J Cereb Blood Flow Metab. 2011;31(5):1243-1250. doi:10.1038/jcbfm.2010.209.

5. Wang M, Zhou C, Yu L, Kong D, Ma W, Lv B, et al. Upregulation of MDH1 acetylation by HDAC6 inhibition protects against oxidative stress-derived neuronal apoptosis following intracerebral hemorrhage. Cell Mol Life Sci. 2022;79(7):356. doi:10.1007/s00018-022-04341-y.

6. Lan X, Han X, Li Q, Wang J. (-)-Epicatechin, a natural flavonoid compound, protects astrocytes against hemoglobin toxicity via Nrf2 and AP-1 signaling pathways. Mol Neurobiol. 2017;54(10):7898-7907. doi:10.1007/s12035-016-0271-y.

7. Cox J, Mann M. MaxQuant enables high peptide identification rates, individualized p.p.b.-range mass accuracies and proteome-wide protein quantification. Nat Biotechnol. 2008;26(12):1367-1372. doi:10.1038/nbt.1511.

8. Padavattan S, Shinagawa T, Hasegawa K, Kumasaka T, Ishii S, Kumarevel T. Structural and functional analyses of nucleosome complexes with mouse histone variants TH2a and TH2b, involved in reprogramming. Biochem Biophys Res Commun. 2015;464(3):929-935. doi:10.1016/j.bbrc.2015.07.070.

9. Li Q, Lan X, Han X, Durham F, Wan J, Weiland A, et al. Microglia-derived interleukin-10 accelerates post-intracerebral hemorrhage hematoma clearance by regulating CD36. Brain Behav Immun. 2021;94:437-457. doi:10.1016/j.bbi.2021.02.001.

10. Shi X, Bai H, Wang J, Wang J, Huang L, He M, et al. Behavioral assessment of sensory, motor, emotion, and cognition in rodent models of intracerebral hemorrhage. Front Neurol. 2021;12:667511. doi:10.3389/fneur.2021.667511.

11. Qu J, Zong HF, Shan Y, Zhang SC, Guan WP, Yang Y, et al. Piezo1 suppression reduces demyelination after intracerebral hemorrhage. Neural Regen Res. 2023;18(8):1750-1756. doi:10.4103/1673-5374.361531.

12. Li Q, Weiland A, Chen X, Lan X, Han X, Durham F, et al. Ultrastructural characteristics of neuronal death and white matter injury in mouse brain tissues after intracerebral hemorrhage: coexistence of ferroptosis, autophagy, and necrosis. Front Neurol. 2018;9:581. doi:10.3389/fneur.2018.00581.

13. Kaya-Okur HS, Wu SJ, Codomo CA, Pledger ES, Bryson TD, Henikoff JG, et al. CUT&Tag for efficient epigenomic profiling of small samples and single cells. Nat Commun. 2019;10(1):1930. doi:10.1038/s41467-019-09982-5.

# Supplementary Tables

**Table S1. Antibodies used in this study**

| **Antibody** | **Application** | **Supplier** | **Catalog No.** |
| --- | --- | --- | --- |
| Histone H2B | Immunoprecipitation; immunoblotting; CUT&Tag | Abcam | ab1790 |
| Acetyl-lysine | Immunoblotting | Cell Signaling Technology | 9441 |
| BCL2 | Immunoblotting | Abcam | ab182858 |
| BAX | Immunoblotting | Abcam | ab32503 |
| GALC | Immunoblotting | Proteintech | 11991-1-AP |
| Myelin basic protein | Immunofluorescence | Cell Signaling Technology | 83683 |
| Neurofilament 200 | Immunofluorescence | Cell Signaling Technology | 2836 |
| Flag | Immunoprecipitation | Proteintech | 20543-1-AP |
| β-tubulin | Immunoblotting | Cell Signaling Technology | 2128 |

**Table S2. Primer sequences used in this study**

| **Gene** | **Forward primer (5′-3′)** | **Reverse primer (5′-3′)** |
| --- | --- | --- |
| GAPDH | CTGGAGAAACCTGCCAAGTA | TGTTGCTGTAGCCGTATTCA |
| TNF | CGCTCTTCTGTCTACTGAACTTCGG | GTGGTTTGTGAGTGTGAGGGTCTG |
| IL-6 | CTTCTTGGGACTGATGCTGGTGAC | TCTGTTGGGAGTGGTATCCTCTGTG |
| IL-1β | GCAACTGTTCCTGAACTCAACT | ATCTTTTGGGGTCCGTCAACT |
| CD86 | TGTTTCCGTGGAGACGCAAG | TTGAGCCTTTGTAAATGGGCA |
| NLRP3 | GGACAGCCTTGAAGAAGAGTGGATG | CTGCGTGTAGCGACTGTTGAGG |
| TGF-β | CATTGCTGGTCCAGTCTGCTTCG | TGGTGAATGACAGTGCGGTTATGG |
| IL-10 | GCTCTTACTGACTGGCATGAG | CGCAGCTCTAGGAGCATGTG |
| ARG1 | ATGCTCACACTGACATCAACACTCC | GGTCTACGTCTCGCAAGCCAATG |
| IL-4 | TACCAGGAGCCATATCCACGGATG | TGTGGTGTTCTTCGTTGCTGTGAG |
| GALC | CTGGATACTCTATGGCTCCTTGAC | AGTGGTGAGCGTAAATATCTCGTC |

# Supplementary Figure Legends

**Figure S1. Experimental localization and acetylome quality-control information.**

(A) Stereotaxic surface coordinates. (B) Injection depth and the hematoma, perihematomal, CPu, and CC analysis regions. (C) Coomassie-stained, histone-enriched brain extracts used for acetylome profiling (day-1 sham and day-1 ICH; n=3/group). (D) Validation of the H2B immunoprecipitation/acetyl-lysine immunoblotting workflow using input, IgG, and H2B-IP fractions. (E) Numbers of identified and quantified acetylated proteins, peptides, and sites. (F) Heat map of quantified acetylation sites, with H2BK109ac indicated.

**Figure S2. Inflammation-related transcripts and complete RNA-seq enrichment results.**

(A) Tnf, Il6, Il1b, Cd86, and Nlrp3 transcripts. (B) Tgfb, Il4, Il10, and Arg1 transcripts in NC, H2BK109R, and H2BK109Q brain tissue on day 1 (n=6/group). (C) KEGG enrichment of mRNAs significantly upregulated in the brains of H2BK109Q mice compared with H2BK109R mice, and (D) Gene Ontology biological process, cellular component, and molecular function results for these significantly upregulated mRNAs. (E) Detailed enrichment maps of these significantly upregulated mRNAs in glutamatergic, cholinergic, serotonergic, and GABAergic synapse pathways. Data are presented as mean ± SD. *P<0.05, **P<0.01, ***P<0.001, ****P<0.0001.

**Figure S3. Additional histological and TEM measurements.**

(A) H&E-stained whole coronal sections and enlarged CC and CPu regions from Con, NC, H2BK109R, and H2BK109Q mice (Scale bar=625 μm; enlarged images, scale bar=100 μm). (B) Additional NF200/GFP images in NC, H2BK109R, and H2BK109Q tissue (Scale bar=20 μm; enlarged images, scale bar=10 μm). (C) TEM-derived myelin thickness in NC, H2BK109R, and H2BK109Q mice (n=3 mice/group). (D) Myelin thickness after siNC or siGALC delivery in H2BK109Q mice (n=3 mice/group; 72 H2BK109Q, 74 K109Q-siNC, and 73 K109Q-siGALC axons). Symbols above D denote Holm-adjusted comparisons of mixed-effects-model slopes for myelin thickness versus axon diameter: H2BK109Q versus K109Q-siNC, P=0.3781; H2BK109Q versus K109Q-siGALC, P=0.0206; K109Q-siNC versus K109Q-siGALC, P=0.0023. Axons were nested within mice and were not treated as independent biological replicates. *P<0.05, **P<0.01; ns, not significant.

**Figure S4. Supplementary metabolomic and total-H2B CUT&Tag results.**

(A-E) Principal component analysis, volcano plot, metabolite heat map, KEGG summary, and sphingolipid pathway map for H2BK109Q and H2BK109R HT22 cells under the same Hb challenge (n=3/group). (F) CUT&Tag peak Venn diagram and global total-H2B signal.
